# Application of a High-Precision Aeolian Sand Collector in Field Wind and Sand Surveys

**DOI:** 10.3390/ijerph18147393

**Published:** 2021-07-10

**Authors:** Xinchun Liu, Yongde Kang, Hongna Chen, Hui Lu

**Affiliations:** 1 Institute of Desert Meteorology, CMA, Taklimakan Desert Meteorology Field Experiment Station of CMA, Xinjiang Laboratory of Tree Ring Ecology, Key Laboratory of Tree-Ring Physical and Chemical Research of China Meteorological Administration, Urumqi 830002, China; liuxch@idm.cn; 2 State Key Laboratory of Eco-Hydraulics in Northwest Arid Region of China, School of Water Resources and Hydroelectric Engineering, Xi’an University of Technology, Xi’an 710048, China; 3 Urumqi Environmental Monitoring Center, Urumqi 830001, China; liuxinchun2001@163.com; 4 Key Laboratory of Ecology of Rare and Endangered Species and Environmental Protection, Guangxi Normal University, Ministry of Education, Guilin 541004, China; luhui1008@163.com; 5 College of Environment and Resources, Guangxi Normal University, Guilin 541004, China

**Keywords:** soil erosion, wind erosion, sand collection efficiency, dust horizontal flux

## Abstract

Sand collectors are important for quantitatively monitoring aeolian sand activities. In this paper, an automatic high-precision sand collector was designed. Based on the measured data of aeolian transport performed with a piezoelectric saltation sensor (H11-Sensit) and a 10 m high meteorological tower, the sampling efficiency of the automatic sand sampler and the horizontal dust flux of the near surface were analyzed based on observed data. The results were as follows: the best-fitting function between the number of impacting sand particles and the amount of collected sand was a linear relationship. The average value of *R*^2^ was 0.7702, and the average sand collection efficiency of the sand collector at a height of 5 cm was 94.3%, indicating good sand collection performance. From all field tests conducted so far, it appeared that a high-precision sand sampler was a useful device for making field measurements of horizontal dust fluxes and ascertaining the relationship between transition particles and wind speed. In the future, the equipment costs and wind drive will continue to be optimized.

## 1. Introduction

Wind is an important erosion force that shapes the surfaces of Earth, Venus, Mars, and Titan [1,2]. In arid, semi-arid, high latitude, and high-altitude regions on Earth, wind erosion is usually considered to be the main driving force for soil loss and dust release [3,4,5,6]. Quantitative studies of wind-induced sand migration have played an important role in revealing the geomorphological processes, and soil losses and wind erosion dust have been accurately simulated [7,8,9]. In some countries, the annual average particulate matter (e.g., PM2.5 and PM10) mass concentrations are high [10], where the major emission sources of PM are the degradation of soil and the mismanagement of lands [10].

Many studies have been carried out on horizontal sand dust fluxes, such as in Europe, China, Canada, Australia, and the United States, especially in arid and semi-arid regions. On Earth, this occurs mainly in deserts, on beaches, and in other sparsely vegetated areas, such as dry lake beds [11,12,13,14,15]. The lift-off velocities of differently sized particles obey different distribution functions. The mean particle velocity at different heights also depends on momentum exchange and particle concentration.

Because of the difficulty of obtaining relevant measurements, integrated dust samplers have historically been the most common method for measuring sand dust fluxes in the field and in laboratory investigations [16,17,18]. Different researchers have designed various types of sand samplers [19,20,21,22] to study the horizontal and vertical fluxes of dust transport in the near strata [23,24,25,26,27,28,29,30,31,32,33,34,35], and they are widely used to obtain sand transport measurements in field and in wind tunnel experiments [36,37,38,39]. Initial designs were improved by increasing the sampling efficiency through taking account of the aerodynamics associated with blocking a portion of the flow [17]. Additional improvements were made by increasing the temporal resolution through automatic weighing systems [40,41] to retroactively apply a temporal signature to the mass flux of sand dust [42]. However, the spatial and temporal resolution of a mass-collecting sand dust traps remain insufficient for capturing most small-scale aeolian processes. In addition, because sand traps obstruct aerodynamic processes to varying degrees, the efficiency of sampling sand dust is variable, and differs with height and wind conditions [43], but is generally around 80% [44]. In short, traditional sediment collectors (such as the big spring number eight, BSNE, and modified Wilson and Cooke (MWAC) samplers) cannot automatically collect and weigh sediment flux, nor can wind erosion sensors (such as the Sensit and Wenglor laser fork).

The aim of this study was to understand the sand dust transport process and sampling efficiency more fully. Thus, our research group designed an automatic, long-period, and high-frequency sand sampler. The sand collector designed in this paper has two innovations, focused at overcoming two problems: one is the interference of the sand collector on the airflow in the measurement section and the other is the exhaust problem inside the sand collector. The inlet of the sand sampler has been designed as a wedge, and a wind speed guide groove and airflow guide outlet are also incorporated into the design. A hollow shaft was designed as a special dustproof system (i.e., with first and second air leakage networks) so that the airflow could be discharged into the atmosphere before entering the hollow shaft. Consequently, the influence of the sand collector on the airflow was minimized, which conformed to the principle of equal dynamic performance of the sand collector design, which is of great significance to improving the sand collection efficiency. Additionally, the dust collection of the sand collector realized the real-time dynamic monitoring of wind erosion by combining dynamic dust collection with static weighing. Through this multistage weighing approach, a historic breakthrough was achieved in terms of measurement accuracy and resolution, and the measurement range has been greatly improved. Thus, the high-precision, high-frequency, and long-period automatic monitoring of sand dust was truly realized, which automatically recorded at high speeds and accurately reflected any changes of the dust. In addition, the advantages of this sand collector were a low failure rate, strong maintainability, and a wide range of use. It was not only able to observe the wind and sediment activities at the height of several centimeters to several meters above the surface, but was also able to observe these at heights of zero to several hundred meters.

Field experiments were conducted using the sand sampler, existing meteorological data, and a Sensit wind erosion sensor to reveal the characteristics of the mass flux, mass concentration, horizontal velocity, and impact number of particles, thus enhancing our understanding of the characteristics and relationships of dust activities in the area. At the same time, the results from this paper provided useful input data for theoretical analyses and numerical model validation.

## 2. Materials and Methods

### 2.1. Study Area

**Figure 1:**
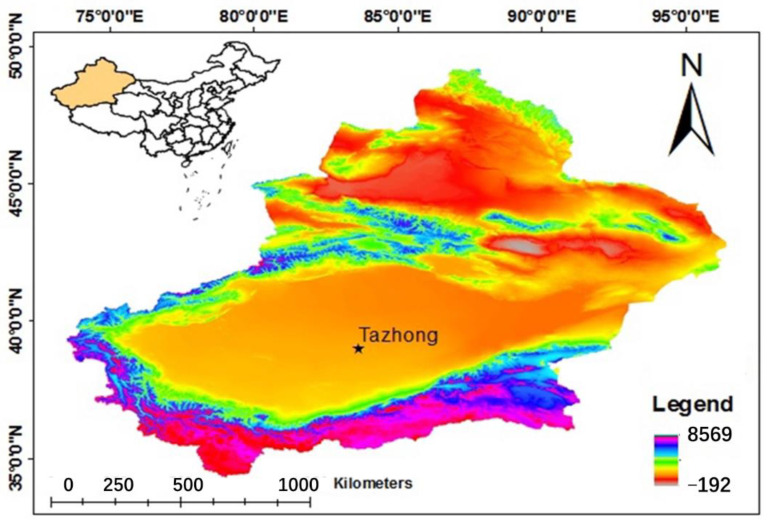
Map of the study area, showing the location of the Tazhong site in the Taklimakan Desert of Xinjiang Province, China.

The study area is located around a western station used to make wind drift sand flow observations in Tazhong, China (83°39′ E, 38°58′ N), in the hinterland of the Taklimakan Desert (Figure 1). The area has a typical temperate continental climate, with high temperatures in dry summers, including little precipitation, rich sand resources, and scarce vegetation. It has an average annual temperature of 13.6 °C, temperature extremes of 46.0 °C and −5.0 °C, an average annual precipitation of only 25.9 mm, and an average annual evaporation capacity of up to 3812.3 mm. The area is also characterized by frequent blowing sands and sandstorms, with more than 500 annual sand windstorms (6.0 m/s, measured at 11.4 m above the ground), average wind speeds of 2.5 m/s, a maximum instantaneous wind speed of 20.0 m/s, and a blown-sand activity index of about 8000. The main wind directions are ENE, EN, and NNE. The frequency of sandstorms and blowing sands was high from March to August. The regional sandstorms in this area feature long durations and poor visibilities. Previous studies showed that 91% of the sand grains at a 5 cm height were in the range of 63–250 μm, with most in the range of 125–250 μm [45,46].

### 2.2. Construction of the Sand Collector

**Figure 2:**
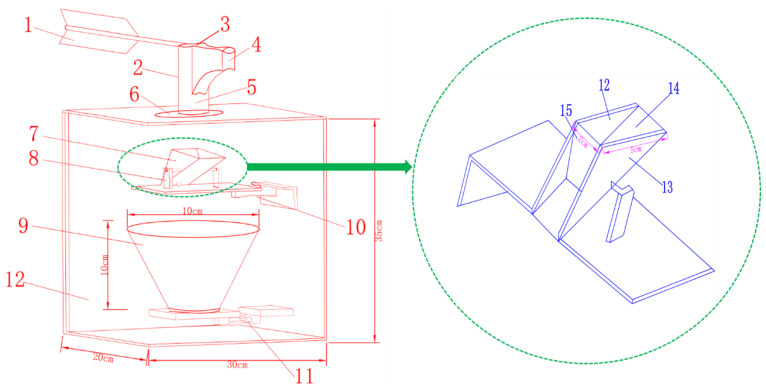
Structure diagram of the fully automated high-precision sand collector: 1. empennage; 2. first air leakage network; 3. second air leakage network; 4. sand intake valve; 5. hollow shaft; 6. bearing dust proof seat; 7. small-capacity container; 8. container support shaft; 9. large-capacity container; 10. small-range, high-precision weighing sensor; 11. large-scale weighing sensor; 12. first triangular plate; 13. second triangular plate; 14. first rectangular plate; 15. second rectangular plate.

The core structures of the sand sampler (Figure 2) were a dust collection system and a dust measurement system. The dust collection system was installed above the device enclosure (components 1–6 in Figure 2), which mainly consisted of an empennage, first air leakage network, second air leakage network, sand intake, hollow shaft, and bearing dust proof seat. The static measurement system was in the device enclosure, which was mainly composed of a small-range, high-precision dust weighting system and a large-scale dust weighting system. The small-range, high-precision dust weighting system consisted of an automatic sand release device and a small-scale, high-precision weighing sensor. The small-scale, high-precision weighting sensor was mounted on the inner wall of the device enclosure, and the automatic sand release tipping bucket was related to the small-scale, high-precision weighting sensor. The automatic sand release device consisted of a small-capacity container (tipping bucket) and a container support shaft. The small capacity container and container support shaft were related to a flexible rod, which can rotate around the support shaft. The large-scale dust weighting system consisted of a large capacity container and a large-scale weighting sensor. The large capacity container was mounted on the second platform. One end of the large-scale weighting sensor was fixed under the second platform and the other end was fixed on the inner wall of the device enclosure. Conveniently, the large-capacity container was designed as an inverted truncated cone-shaped container, which could help concentrate the center of the gravity of the collected objects to the sensing part of the weighting sensor.

To improve the measurement accuracy and range of the sand sampler, graded weighting was adopted. The range of the first-grade weighting container was 0–300 g with an accuracy of 20 mg, and the range of the second-grade weighting container was 0–10 kg. Sampling was carried out with the created device for twelve months at the test site, and automatic observations were realized, where an aspect sensor was used to monitor the sand amounts arising from each direction. The data acquisition system had a high storage capacity (2 GB) and high frequency (≥1 Hz), which allowed us to dynamically and comprehensively record the processes and dynamic variations of dust emission arising from wind erosion in real time and weigh the collected dust with high precision over a wide range.

During the tests, the empennage kept the sand intake rate consistent with the wind direction according to the wind force in the wind direction. At this point, the surface sands entered the hollow shaft through the sand intake valve. Sand-carrying gas flows could be discharged from the first air and second air leakage networks. Sands then automatically sank to the small-capacity container via gravity, and the sand weighting sensor began to sense (accuracy: 20 mg). The weight data were subsequently uploaded to the data acquisition system. Then, the aspect sensor uploaded the aspect data of the acquired sand to the data acquisition system to obtain the instantaneous sand amount in the corresponding direction. If the collected sands were over 240 g, the tipping bucket rotated automatically to pour the sands into the large-capacity container for weighing (weight range: 0–10 kg). Not only was the total sand amount in the whole process recorded, but also the data measured by the small capacity sensor were calibrated, realizing long-term automatic monitoring, and reducing the manual workload. The data acquisition system was connected to a GPRS/CDMA communication module and power supply system, and the GPRS/CDMA communication module was wirelessly connected with the computer, with which real-time online observations were achieved offsite or onsite. The observation frequency was up to 10 Hz. All the data measured during an experiment, including wind speed, wind direction, sand transport aspect, instantaneous aspect sand flux, profile sand flux, and cumulative total sand flux, were uploaded to the data acquisition system. Through the wireless transmission network, the backstage carried out real-time remote automatic monitoring of the acquired data.

### 2.3. Data Collection and Processing

**Figure 3:**
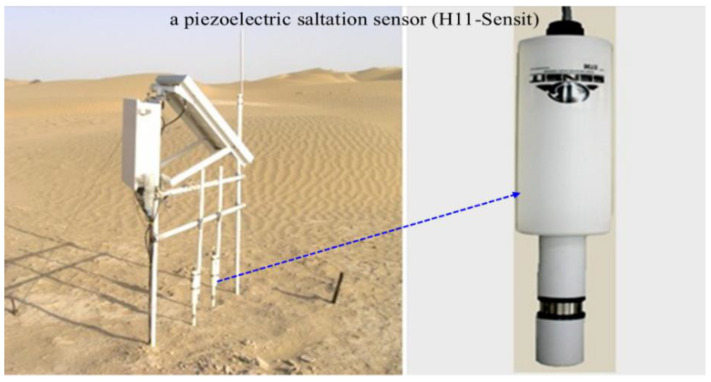
Wind erosion sensor (H11-Sensit).

Fifteen dust events from July to August in 2010 were studied. Blowing sands and sandstorms are frequent in this area, which facilitated the collection of experimental data. The automatic sand sampler developed by [46] (Figure 2) was used, which had a sand intake area of 5 cm × 2 cm and a collection frequency of 1 Hz. An H11-B wind erosion sensor (H11-Sensit, American SENSIT Company: Valparaiso, IN, USA) (Figure 3) was used to record the number of sand particles impacting the sensor every second. The sensor probe was installed 5 cm above the ground. The sand sampler was mounted on a horizontal sand surface with a straight-line distance of 50 m from the wind erosion sensor to ensure that the sand sampler empennage could rotate normally on windy days. The sand intake height of the sand sampler was the same as the height of the sensor probe. A national standard test sieve was used for statistics.

The average particle size of sands was measured to be 63–250 μm. Sands with particle sizes greater than 74 μm accounted for 75.57%, and those with particle sizes greater than 50 μm accounted for 97.65%. The average of the two was 86.61%. The actual sand collection multiplied by 86.61% was the effective weight corresponding to the number of sand particles impacting the sensor. After the experiment, the SigmaPlot12.5 software was used for data processing and plotting.

Yang and He showed that the number of sands particles impinging the sensor (N) and the dust horizontal flux (F) have a linear relationship:

(1) F=0.0512N

At the same time, the sand collection of the BSNE sand sampler at 5 cm above the ground (*y*) and the number of sand particles impacting the sensor (*x*) also had a linear relationship:

(2) y=0.0002x

Equations (1) and (2) were combined to obtain the following equation:

(3) F=256M

where *M* is the mass of sand collected within 5 cm above the ground (units: kg), *N* is the number of sand particles impacting the sensor (in units of 10^3^), and *F* is the horizontal flux of dust passing through the 5 cm × 2 cm section (units: kg).

In our experiments, the number of sand particles impacting the sensor and the mass of collected sand were found to have a linear relationship, as found by [47,48] and described by Equation (2). Therefore, the dust horizontal flux in this paper was calculated using Equation (3). Next, the sand collection efficiency is the ratio of the amount of sand collected by the sand sampler over the actual amount of transported sand. The correlation coefficient of the two was taken here as the average sand collection efficiency.

## 3. Results and Analysis

### 3.1. Analysis of the Correlation between the Number of Impacted Particles and the Amount of Sediment Collected

**Figure 4:**
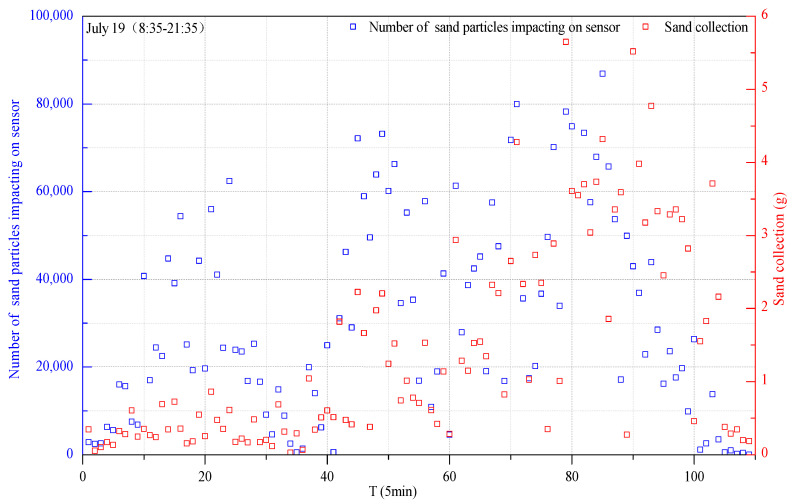

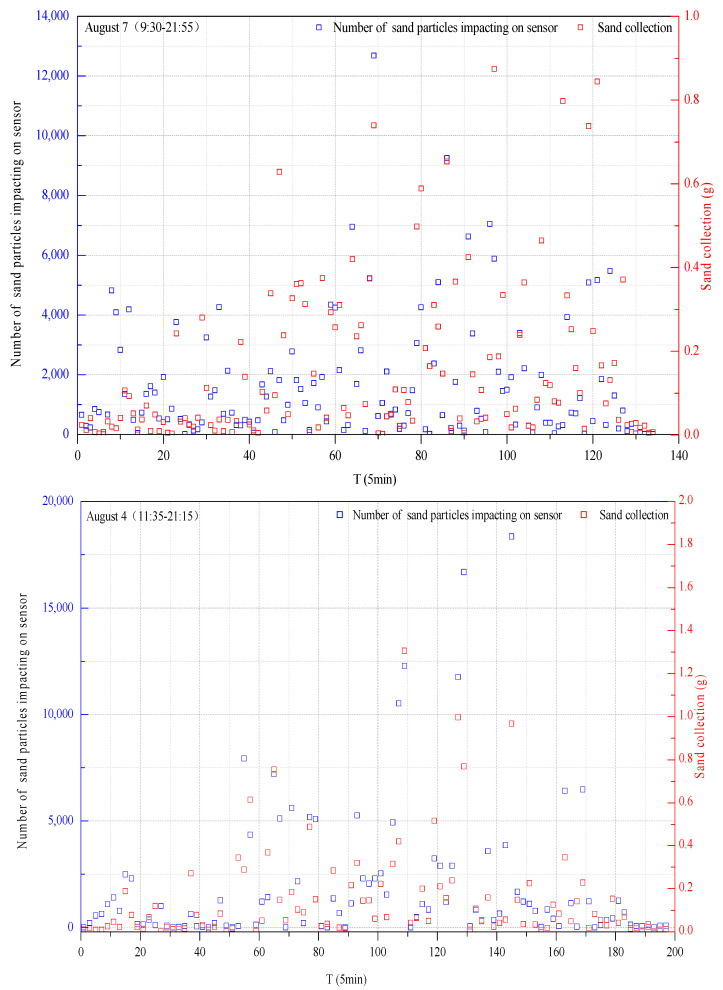

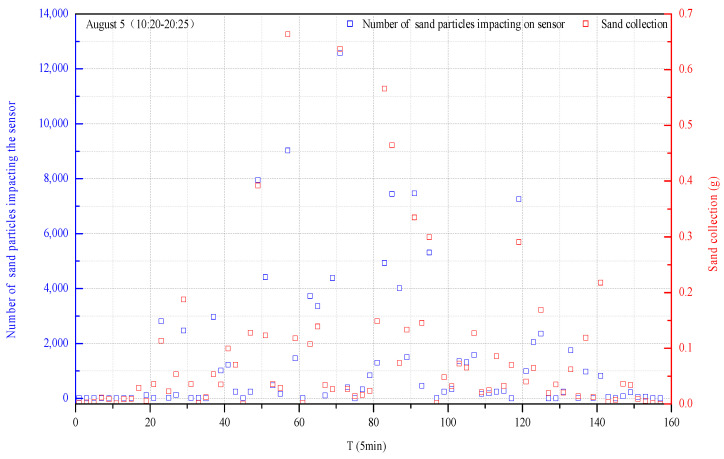
Trends in the number of impact particles and the amount of sediment collected.

Fifteen typical sand–dust weather events from July to August 2015 were monitored to analyze the variation trend of the impacting particle number and sediment concentration. It was found that the variation trend was consistent between sand–dust storms and sand-blown weather, especially on 19 July 2015 (8:35–21:35), 4 August 2015 (11:35–21:15), 5 August 2015 (10:20–20:25), and 7 August 2015 (9:30–21:55) (Figure 4). It was inferred that the variation trend of the impact particle number and sediment concentration was almost consistent for any kind of sand–dust weather, indicating that the field sediment collection performance of the automatic high-precision sand collector was good for both sand–dust storm weather and sand-blown weather.

**Figure 5:**
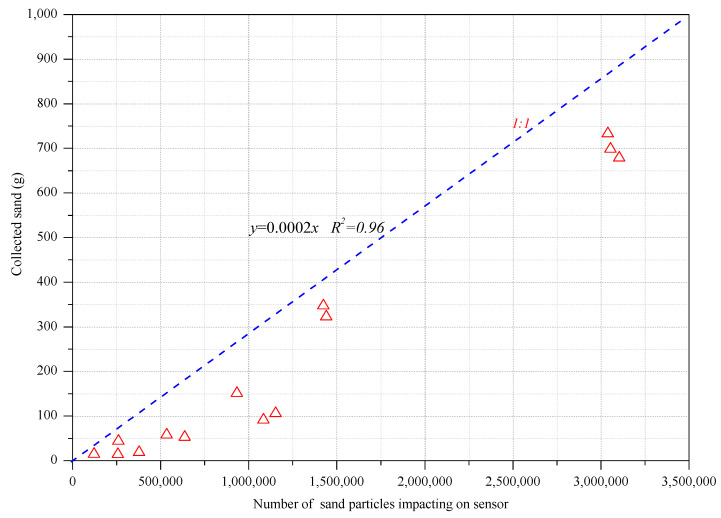
Correlation between the total impact particle number and total sediment concentration.

Figure 5 shows the relationship between the total number of impacting particles and the total sediment concentration corresponding to the 15 dust weather events. It was found that there is a statistically significant linear relationship between the total number of impact particles and the total sediment concentration, where the correlation coefficient, *R*, reached 0.9608. If the sand sampler failed for a short while, the linear equation *y* = 0.0002*x* was used to estimate the sediment concentration at a height of 5 cm in the corresponding time using the number of particles impacting the sensor, so as to verify the reliability of the weighing.

A regression analysis was carried out on the treated sediment concentration data and impacting particle number data by fitting an exponential function, logarithmic function, power function, and linear function to the data. The best-fitting results are shown in Table 1. From the 15 sets of fitting results, the fitting degrees of the linear function and power function were higher than those of the logarithmic function and exponential function. The linear function has 11 times higher results than the power function, and the average was higher than the power function. Therefore, the linear function could better describe the correlation between the number of sand particles impacting and the sediment concentration; that is, the optimal relationship between the number of sand particles impacting and the sediment concentration during each five-minute period was linear.

According to the linear regression analysis of the number of sand particles impacting every 5 min and the amount of sand collected during this period, it was found that the average correlation coefficient *R* is 0.7702. According to the judgment standard, 0.3 < |*R*| < 0.5 is referred to as a weak correlation, 0.5 < |*R*| < 0.8 is referred to as a significant correlation, and 0.8 < |*R*| < 1 is referred to as high correlation. Thus, it can be seen that among the 15 fitting results, nine were significantly correlated, and six were highly correlated. This showed that the linear correlation between the number of sand particles impacting the sensor and the sediment mass was good.

From an energy point of view, the energy gained by impacting particles during wind-blown sand events is extremely complex, especially in the field, where the impacting particles generally acquire mechanical energy and horizontal kinetic energy in the airflow. According to the conservation of energy theorem, the amount of energy lost by the mass should yield reliable and accurate experimental results.

### 3.2. Sand Collection and Sand Collection Efficiency

**Figure 6:**
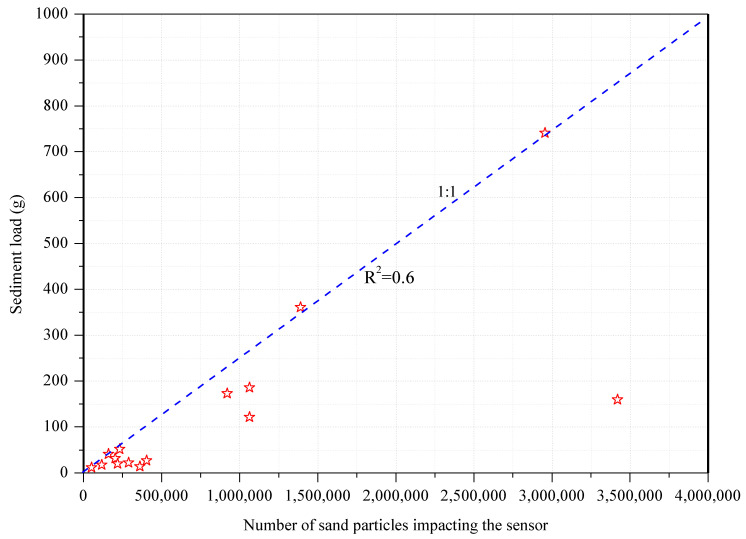
Change of sand collection quantity with saltation particle numbers.

The number of sand particles impacting the sensor during the 15 dust events and the theoretical values of the collected sand masses in Table 2 were fitted, and, as before, the moving particles in the airflow are approximately equal to the energy lost as they impact the sensor; this energy can be ascertained by monitoring the total energy of the impacting particles per second at a certain height. In different periods of the 15 dust events, different wind drift sand flows were observed, which were influenced by the wind speed and sand source supply condition, which resulted in different fitting functions for the number of sand particles impacting the sensor and the amount of collected sand. Therefore, two or more functions should be combined when studying the relationship between the number of sand particles impacting the sensor, and the collected sand results were well-described by a linear relationship. The coefficient of determination, *R*^2^, was 0.6 (Figure 6). Therefore, the theoretical value of the collected sand mass in each dust event could be calculated according to the fitted linear equation. The calculated results are also shown in Table 2. The statistical results showed that the theoretical values were smaller than the observed values. The reason for this may be that the data described by the linear relationship do not perfectly follow the best-fitting line, but are distributed about it. The field test and wind tunnel test data were different, so the correlation coefficient was not 1 and it was inevitable that the theoretical value of sand collection was smaller than the observed value.

**Figure 7:**
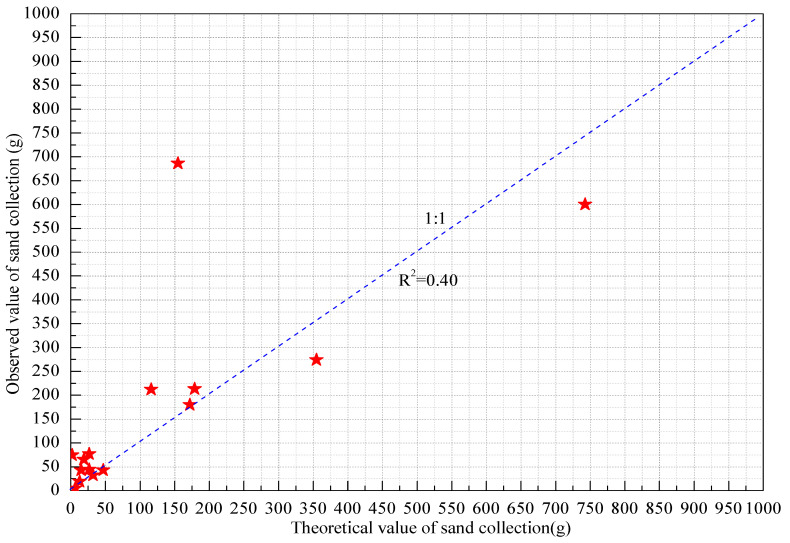
Relationship between theoretical and observed sand masses.

As shown in Figure 7, the theoretical and observed sand masses were fitted and analyzed, and the two did not show any linear relationship, with a *R*^2^ of only 0.40, indicating the large difference between the observed and theoretical values. The reason for this large discrepancy was that the theoretical values were calculated based on the actual amounts of collected sand and the number of sand particles impacting the sensor. In addition, the field environment was complex, which resulted in errors of the data acquisition system in the transmission process, which reduced the final mass accuracies.

A similar study found that their active sand sampler had a higher sand collection efficiency than their passive sand sampler in wind tunnel experiments, and found that the sand collection efficiency increased with an increase of grain size [49]. He et al. found that the sand collection efficiency of their active sand sampler was 105% with the error of ±5% [46]. The sand collection efficiency of their passive Leach sand sampler was 85%, and the collection efficiency for sands with particle sizes smaller than 10 μm was 70%. Their Fryrear BSNE sand sampler had a 90% ± 5% collection efficiency for aeolian sands and a 40% collection efficiency for sands with particle sizes smaller than 10 μm. Goossens et al. found that their same sand sampler had a higher efficiency for collecting aeolian sands than for collecting dust [47,48,49], where the larger the size of the collected particles, the higher the collection efficiency of the sand sampler. Therefore, even for the same type of sand samplers, the sand collection efficiency may be different due to different test methods. For example, one experiment showed that the BSNE sand sampler had a sand collection efficiency of 100–120% [48], while Xu et al. concluded that their BSNE sand sampler had a sand collection efficiency of 90% [49]. Although it was impossible to calculate the sand collection efficiency of each dust event directly based on the observed sand masses and the theoretical values of the sand masses in this work, the average sand collection efficiency could be calculated according to the correlation coefficient between the two: the average sand collection efficiency of the sand sampler at a height of 5 cm was 94.3%. Therefore, the method of testing the efficiency of sand samplers is a problem worth further studying.

### 3.3. Sand Dust Horizontal Flux Calculation

**Figure 8:**
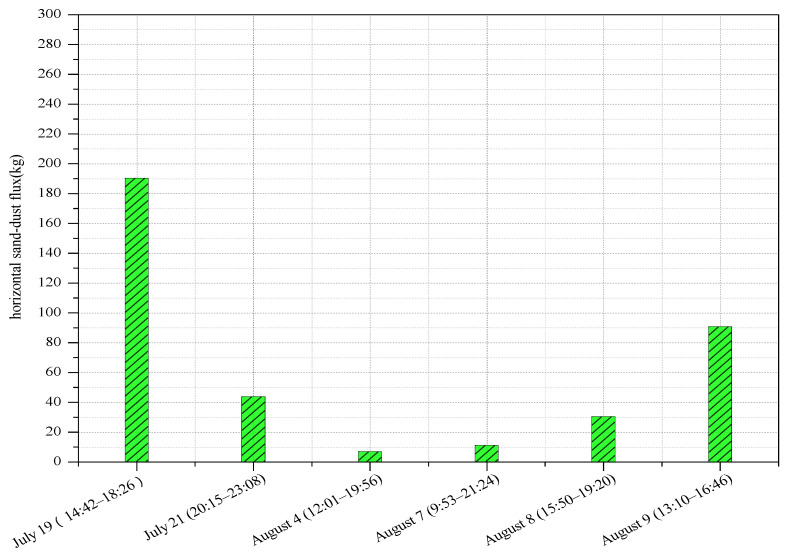
Horizontal sand–dust fluxes on selected dates.

The dust horizontal fluxes were calculated with Equation (3). The variation of particle size and wind speed likely affect the distribution of the dust horizontal fluxes. When the sand particle size was large, the transition height was also high, and the corresponding sand collection flux was also large. It can be seen from Figure 8 that during the 15 dust events, the maximum dust horizontal flux is 190.335 kg, which was measured during a sandstorm. The other events were all blowing sand events, with a minimum value of about 1.2 kg. As these experiments were conducted at the same sampling site, and over a continuous sampling period, the impact of particle size was not taken into consideration in our study; instead, only the impact of wind speed was considered. Unfortunately, more useful information was lost after the wind speeds were averaged. Therefore, the transport of most dust materials happened in the surface layer and they landed on the Earth’s surface by short-distance transportation. Even during a sandstorm, only a small amount of dust was transported at higher altitudes. Thus, when calculating the horizontal dust flux, its functional relationship with height should be established. In this study, the relationship between the collected sand within a height of 5 cm from the ground by the sand sampler and the horizontal dust flux was used; specifically, the former was used to calculate the latter using Equation (3) via a simplified calculation method that reduced the computational requirements.

The dust horizontal fluxes were calculated with Equation (3). The variation of particle size and wind speed likely affect the distribution of the dust horizontal fluxes. When the sand particle size was large, the transition height was also high, and the corresponding sand collection flux was also large. It can be seen from Figure 8 that during the 15 dust events, the maximum dust horizontal flux is 190.335 kg, which was measured during a sandstorm. The other events were all blowing sand events, with a minimum value of about 1.2 kg. As these experiments were conducted at the same sampling site, and over a continuous sampling period, the impact of particle size was not taken into consideration in our study; instead, only the impact of wind speed was considered. Unfortunately, more useful information was lost after the wind speeds were averaged. Therefore, the transport of most dust materials happened in the surface layer and they landed on the Earth’s surface by a short distance.

### 3.4. Sand Transportation Rate and Wind Speed

**Figure 9:**
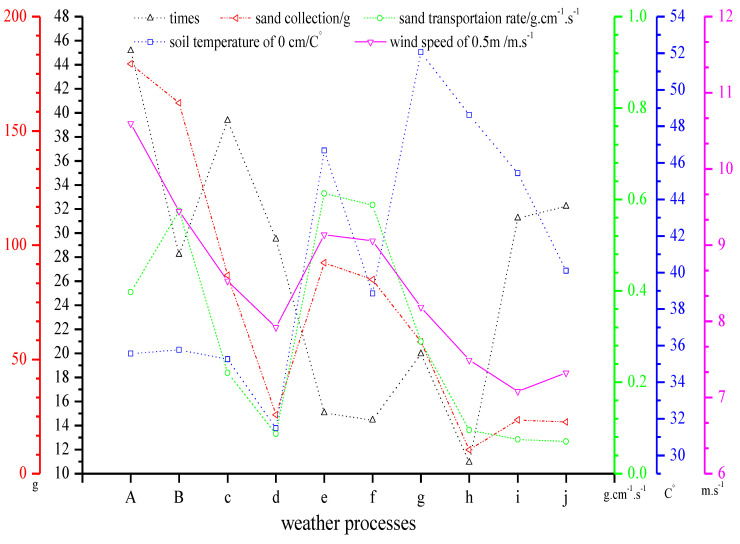
Sediment of different dust events.

Figure 9 shows the relationship between the sand transportation rate and wind speed under different dusty weather conditions; (a) and (b) were strong and typical sandstorm weather events, respectively, and (c) to (j) were blowing sand weather events. In general, the rate of sand–dust transport increased with an increase of wind speed, but the changing trends of the wind speed and sand–dust transport rate during some weather processes were inconsistent. For example, for some blowing sand events (e, g, i, and j) and strong sandstorm events (a and b), although the wind speeds were small, the variations of the sand transportation rate were large. The soil temperature at 0 cm during the corresponding events showed that the soil temperature rapidly increased with an increase of solar radiation intensity. In the surface layer, a strong decreasing temperature structure formed in the surface layer, and the thermal turbulence became stronger and stronger, resulting in enhanced heat transfer and unusually active sand saltation. When the wind speed was small, but the sand transportation rate was relatively large, the corresponding soil temperature at 0 cm was hot. This showed that the increased soil temperature at 0 cm could promote the saltation of sands. This finding is consistent with the results of [48], who found that the “synchronization of wind and temperature” was in favor of the co-occurrence of wind and saltation. The specific relationship of the sand transportation rate with wind speed and temperature needs to be further studied.

## 4. Discussion

There are many factors affecting the horizontal flux of sand dust. In addition to differences in collection instruments, there are many other factors, especially in terms of physical mechanisms, which are summarized in the following.

The movement of sand dust in response to wind is the most important feature of aeolian sediment transport. Wind accelerates particles and ejects more particles when impacting a surface, which leads to increases in the horizontal dust flux in the initial stages [50,51]. However, this rapid increase in the impact particle concentration produces a corresponding increase in the drag of dust particles on the fluid, thereby retarding the wind speed. This in turn reduces the speeds of the horizontal dust flux particles, such that a steady state is reached when the speeds of the impacting particles are reduced to a value at which there is a single particle leaving the soil surface for each particle impacting it [52]. Because of the finite response time of dust flux particle speeds to the wind speed, the horizontal dust flux can “overshoot” the eventual steady-state mass flux [53,54,55].

Kok, Parteli, and Michaels found that the wind profile was modified through momentum transfer by impacting particles [14]. On the one hand, it is the retardation of the wind profile through drag by the saltating particles that ultimately limits the number of particles that can be saltated under given conditions. On the other hand, the drag produced by saltating particles reduces the horizontal momentum flux carried by the wind. At the same time, although the particle speeds at the surface were once thought to remain constant, numerical simulations and wind-tunnel measurements have shown that the particle speeds increase as a function of the shear velocity above the surface. This means that the mass flux higher up in the saltation layer increases relative to that in lower layers, producing a slight increase in the saltation layer height with shear velocity. The extraction of wind momentum by saltating particles produces a steady-state wind profile that accelerates saltating particles to a saltate speed that, on average, results in a single particle leaving the soil bed for each particle impacting upon it.

Rice, Willetts, and McEwan found that, when the surface shear stress fell below the impact threshold, fewer saltate particles were entrained by the wind. This in turn reduced the transfer of momentum from the fluid to the impacting particles, thereby increasing the surface shear stress back to its threshold value. Conversely, when the surface shear stress exceeded the threshold value, more saltating particles were entrained, again restoring the surface shear stress to its critical value. Experiments have indicated that particles with a larger cross-sectional area have a correspondingly higher chance of being “splashed” by a saltate particle [56]. Saltate particles may have short lifetimes because their high inertia causes them to attain lower speeds when splashed, leading them to quickly settle back to the dust surface [57]. Actually, winds cannot directly lift dust particles because the corresponding interparticle cohesive forces are large compared to aerodynamic forces. Instead, these dust particles mainly collide with each other under wind erosion [58,59]. During a dust storm, lifted particles are accelerated by the wind, and the resulting impacts on the dust bed can eject, or splash, new erosion particles into the fluid stream. This process produces an exponential increase in the particle concentration [60], which leads to increasing drag on the wind, thereby retarding the wind speed in the erosion layer [61]. It is this slowing of the wind that acts as a negative feedback process that reduces particle speeds, which ultimately limits the number of impacting particles [62].

Dust flux is predominantly emitted by the saltates of eroding particles at the near surface [58,59]. Several authors have also argued that mid-air collisions can affect particle trajectories and the mass flux at large shear velocities [63,64,65,66]. Wind erosion produced by colliding particles during a single hop is of the order of 10–50%, which increases with wind speed [66]. That is, even at large impact speeds, not all saltating particles rebound from the surface, because some will dig into the particle bed [67]. Moreover, recent measurements indicated that the restitution coefficient is a declining function of the saltator impact speed [68]. Numerical simulations [50,53,69,70], laboratory experiments [71], and theory [57] have all indicated that the number of splashed particles scales with the impacting momentum. Experiments have also indicated that the fraction of the average impacting momentum spent on splashing surface particles is of the order of 15% for a bed of loose sand particles [56,57,59]. Most splashed particles, therefore, move in low-energy reptation trajectories and quickly settle back to the soil bed. However, some splashed particles do gain enough momentum from the wind to participate in saltation and splash up more particles. This multiplication process produces a rapid increase in the particle concentration upon initiation of saltation, where the distribution of splashed speeds at a given ejected velocity follows either an exponential or a lognormal distribution [72,73].

Sand transport has been measured by many instruments developed mainly to determine the rates of transported material. Accurately measuring these processes has been an ongoing challenge since the first known discrete measurements were made by Bagnold. In general, impact sensors suffer from poor sensitivity to small sand grains [74], and in the case of Safire, poor interinstrument repeatability [75]. Over the past decade, although instruments vary in design and complexity, they can be split into two categories: integrating and real-time electronic instruments. As achieved in this study, there have been incremental improvements in this latter category of devices that have been motivated, in part, by the observation that sediment transport occurred on spatial scales smaller than 5 cm and temporal scales shorter than 1 s. Initial designs were improved by increasing the efficiency by taking account of the aerodynamics associated with blocking a portion of the flow [13]. Additional improvements were made by increasing the temporal resolution through automatic weighing systems [27,28]. However, the spatial and temporal resolutions of a mass-collecting sediment trap remain insufficient for capturing most small-scale aeolian processes. In addition, because sand traps obstruct the flow to varying degrees, the efficiency of sampling saltating grains varies with height and wind conditions [17] but is generally around 80% [44]. Sand particle sizes not only differ regionally [76], but also vary significantly with height [77,78,79]. This variation of sand particle size with location and height inevitably influences the sampling efficiency of different samplers, leading to errors in the amount of aeolian sand collected. However, in previous experiments, this has often been neglected, or a fixed sampling efficiency has been applied [48,80,81,82].

Some research has shown that an exchange of sand is expected between adjacent dunes of different heights, since larger dunes are more likely to disperse sand than smaller ones [83]. Changes in individual dunes relative to sand flux are unstable, meaning that in one area, dunes that evolve only through sand flux should eventually merge into larger dunes [84]. It is well-known that dune patterns are mainly determined by the amount of available sand and the behavior of the wind in the year. At the same time, changes in the wind and its direction may destroy the stability of large dunes, thereby affecting sediment flux [85]. However, wind erosion processes are often random or strongly dependent on local environmental conditions, making them difficult to incorporate into general analyses or simulations of dune fields. Additionally, collisions between sand particles can adjust the dune shape and the distance between them. When sand particles collide with each other, sand will be redistributed between the dunes [86]. There is not yet a general theoretical model that can self-consistently explain this diversity of dune patterns.

In order to understand the mechanism of sand–particle collisions between sand dunes, it is necessary to use a continuum model for numerical simulations. Continuum dune models have been used to quantitatively connect dune characteristics to environmental conditions and physical processes, including interactions within a single sand dune and between multiple sand dunes. At larger scales, each sand dune is regarded as a single particle, and the results of the large-scale continuum model are used to explain the collisions between particles, i.e., interactions between sand dunes. The pioneering work of [87] is the basis of most sand dune formation and evolution continuum models. These models have been mainly applied to study the influences of wind and sand flux on details of dune morphology [88,89], and the influence of vegetation and induration in stabilizing dunes [88]. Many studies have examined the characteristic scales of dune formation and evolution in different environments, such as underwater or on Mars [90,91]. Jump particles on the back of a windward dune hit the bottom of the dune, causing sand deposition, and ultimately remain in the windward dune. With the transport of windward dune sand, the dune’s contour becomes shorter, which leads to a speed increase in the sand flux. Similarly, windward dunes accumulate sand, become taller, and thus reduce the sand flux speed [87]. It seems that the size ratio between dunes, not the individual size of dunes, determines the impact of sand collision on sediment transport [92,93,94].

In future studies, we will attempt to apply a continuum model to fields composed of hundreds of dunes. However, due to the large temporal and spatial scales involved and current technical constraints, its feasibility may be limited [87]. In other words, following the methods adopted in other research and the obtained basic parameters, we hope that the future measurements of sand flux in areas between desert sand dunes will help to develop better models for dune fields, based on previous agent-based simulations [83,87,95]. Such models rely on the accurate description of interdune sand transport, which is still poorly known, and should thus constitute a topic of further investigation in future work.

To sum up, in the complex field environment, differences in the horizontal dust fluxes and dust collection efficiencies were caused by differences in the type, accuracy, and sampling periods of the sand samplers, as well as the sand particle size and height. Therefore, in future research, developing a sand sampling device with a high sand collection efficiency that is sensitive to small disturbances to the flow fields in complicated fields is an important requirement to accurately estimate dust fluxes. Secondly, we aim to develop a numerical model, and then calibrate the model through field-measured data, so as to carry out numerical simulations of large-scale sediment flux and dust flux.

## 5. Conclusions

The cumulative collected sand mass and number of sand particles impacting the sensor of the designed sand sampler were highly consistent in the complex field test environment, which had a linear relationship with an *R*^2^ value of 0.6053, reflecting good performance in sand collection. The sand collection efficiency was about 94.3% for the sand sampler, indicating that the sand sampler could effectively monitor the movement of sand particles. The data obtained from field experiments allowed us to deduce a simple formula for calculating the dust horizontal flux: *F* = 256*M*. We also estimated the maximum and minimum dust horizontal flux within a height of 5 cm near the surface of the mobile sand surface in the tower during 15 dust events, finding values of 190.335 kg and 1.2 kg, respectively.

Based on the simultaneous observations of wind speed and temperature in our attempt to analyze the transitional sediment transport rate at a height of 5 cm, it was found that with an increase of wind speed, the sediment transport rate generally increased, but during some weather periods, the sediment transport rate changed with the wind speed. Inconsistent, and in this case, the landmark temperature during the corresponding period is significantly higher.

## Figures and Tables

**Table 1:** Fitting analysis of saltation particle numbers and sand collection quantity.

	Observation Time	Coefficient of Determination (R^2^)			
		Exponential Function	Logarithmic Function	Power Function	Linear Function
July	19 (8:35–21:25)	0.45	0.62	0.69	0.6
	20 (19:35–21:25)	0.33	0.18	0.26	0.28
	21 (20:20–22day5:25)	0.32	0.34	0.45	0.60
	29 (22:15–30 day 2:15)	0.44	0.3	0.55	0.69
	31 (13:10–8.1 day 7:10)	0.34	0.25	0.35	0.49
August	1 (21:40–23:25)	0.63	0.68	0.75	0.82
	4 (11:35–21:15)	0.44	0.31	0.43	0.72
	5 (10:20–20:25)	0.32	0.38	0.53	0.78
	7 (9:30–21:55)	0.26	0.24	0.25	0.38
	8 (10:35–20:25)	0.28	0.48	0.55	0.44
	9 (13:15–18:05)	0.60	0.31	0.44	0.84
	13 (10:30–21:25)	0.33	0.27	0.32	0.63
	14 (10:00–21:15)	0.43	0.31	0.64	0.47
	18 (10:50–20:50)	0.41	0.21	0.68	0.58
	20 (13:40–19:55)	0.54	0.29	0.56	0.75
	Average	0.41	0.34	0.50	0.61

**Table 2:** Observed saltation particle numbers and observed and theoretical sand masses.

	Observation Time	Impact Number of Particles	Observed Value of Sediment/g	Theoretical Value of Sediment Quantity/g
July	19 (8:35–21:25)	2,936,905	743.4966	587.3810
	20 (19:35–21:25)	3,407,661	154.126	681.5322
	21 (20:20–22 day 5:25)	920,676	170.5492	184.1352
	29 (22:15–30 day 2:15)	147,786	34.6005	29.5572
	31 (13:10–1 day 7:10)	1,050,073	179.586	210.0146
August	1 (21:40–23:25)	19,772	4.5764	3.9544
	4 (11:35–21:15)	195,696	26.6128	39.1392
	5 (10:20–20:25)	116,005	12.3933	23.201
	7 (9:30–21:55)	231,534	43.2746	46.3068
	8 (10:35–20:25)	1,048,891	118.5805	209.7782
	9 (13:15–18:05)	1,380,718	354.3815	276.1436
	13 (10:30–21:25)	210,702	14.2759	42.1404
	14 (10:00–21:15)	390,274	25.0633	78.0548
	18 (10:50–20:50)	293,093	15.7758	58.6186
	20 (13:40–19:55)	349,610	4.5021	69.922

## Data Availability

Not applicable.
